# Opioid Dosage Levels, Concurrent Risk Factors and Self-Perceptions among Chronic Pain, Opioid-Managed Individuals at Elevated Risk for Opioid Overdose

**DOI:** 10.3390/ph14121279

**Published:** 2021-12-08

**Authors:** Matthew S. Ellis, Zachary A. Kasper, Mark Gold, Theodore J. Cicero

**Affiliations:** Department of Psychiatry, Campus Box 8134, St. Louis School of Medicine, Washington University in St. Louis, 660 S. Euclid Ave., St. Louis, MO 63110, USA; zkasper@wustl.edu (Z.A.K.); mgold9876@wustl.edu (M.G.); cicerot@wustl.edu (T.J.C.)

**Keywords:** chronic pain, opioid therapy, prescription opioids, opioid overdose, opioid dosage

## Abstract

While current opioid prescribing guidelines highlight a dose-response relationship between therapeutic management and overdose risk, other concurrent risk factors have also been identified. However, there is little data in assessing the relationship between risk factor prevalence, associated provider communication, and subsequent perceptions of overdose risk among chronic pain, opioid-managed (CPOM) patients. An online questionnaire was distributed in June 2020 to a sample of CPOM individuals (n = 190) treated with an opioid prescription at or above 50 daily MME, or any dosage alongside benzodiazepines. CPOM individuals reported a mean daily MME of 470, with half (52.6%) receiving a concurrent benzodiazepine prescription. All patients reported past month alcohol use, and 67.4% indicated a risk-elevating diagnosed medical condition. In assessing provider communication, 41.6% reported no discussion focusing on the risks of one’s opioid therapy. Subsequently, 62.1% perceived themselves as having “no risk”, and 60.0% were “not at all concerned” (60.0%) about experiencing an opioid overdose. Organizational policies should focus on implementing consistent methods of patient education regarding overdose risk, as well as assessments of behaviors or characteristics that my increase an individual’s risk of opioid overdose. These policies should also include other forms of evidence-based overdose risk prevention such as co-prescriptions of naloxone.

## 1. Introduction

Drug overdose fatalities continue to be a primary public health concern in the United States, with over 93,000 deaths attributed to a drug overdose in 2020, the highest number ever reported [1]. Opioids remain the largest contributor to overdose fatalities. However, while much of the recent focus has been on the influx of illicitly manufactured fentanyl and heroin, which carry greater potency and lethality, prescription opioid analgesics continue to make up a significant proportion of opioid overdose fatalities. In 2019, the Centers for Disease Control and Prevention (CDC) estimated 38 deaths a day attributable to prescription opioids [2].

In order to mitigate the risk of overdose from prescription opioid analgesics and encourage evidence-based, responsible opioid prescribing, the CDC released the “CDC Guideline for Prescribing Opioids for Chronic Pain”. [3] For the approximately 25 million Americans experiencing moderate or severe chronic pain [4], the CDC Guidelines recommends nonpharmacologic or nonopioid pharmacologic treatments for opioid-naive patients, rather than opioid analgesics. However, while evidence suggests decreases in opioid prescriptions following publication of the CDC Guidelines [5,6,7,8], dispensed opioids remain high in the United States, at a rate of 43.3 prescriptions per 100 people in 2020 [9]. In addition, in 2019, according to the National Survey on Drug Use and Health (NSUDH), 9.7 million Americans misused prescription opioid pain medications, further reinforcing the risks these substances pose to public health [10].

The CDC Guidelines also highlight evidence which suggests a dose-response relationship between prescribed opioid analgesics and risk of opioid overdose. Primarily, a daily opioid dosage of 50–100 morphine milligram equivalents (MME) was found to increase the risk of overdose by factors of 1.9–4.6; which increased to 2.0–8.9 for daily dosages greater than 100 MME per day [3,11,12,13]. This led the CDC to recommend against prescribing daily doses greater than 50 MME to opioid-naïve individuals with chronic pain. However, there are several complicating factors related to opioid dosage schedules. Specifically, tolerance to the analgesic effects of opioids can escalate rapidly and is very robust, which may result in the need for doses many times greater than initial ones to achieve pain relief in long-term opioid therapy patients. On the other hand, tolerance to the respiratory effects of opioids is much more modest [14]. Thus, an increase in a dosage for pain can subsequently increase the risk for fatal respiratory depression. This poor therapeutic index (i.e., toxic doses divided by therapeutic doses) can worsen over time, further increasing the risk of unintentional overdose in otherwise medication-compliant patients, particularly when taking into consideration other risk factors that may exist, particularly those that may increase the risk of respiratory depression. In addition, recent research has suggested that hyperalgesia, increased sensitivity to pain, may result from long-term opioid therapy and may result in patients needing greater dosages of opioid analgesics to achieve pain relief equivalent to what was experienced with earlier, lower dosages [15].

Other than dosage recommendations, the CDC identifies a number of extraneous factors that may put patients at further risk of an opioid overdose event. In particular benzodiazepines, which can increase the risk of respiratory depression [11,16,17,18,19,20,21], should be avoided at any dosage alongside a prescribed opioid analgesic. In 2017, one-fifth of US patients with an opioid prescription had at least one day of overlapping opioid and benzodiazepine exposure [5]. A third of opioid overdose fatalities in 2017–2018 involved a benzodiazepine [18], and in 2020 92.7% of benzodiazepine involved overdose deaths also involved an opioid [22]. Benzodiazepines are frequently prescribed during opioid therapy to mitigate sleep-related issues and issues related to mental health, the latter of which is common among patients with chronic pain [23], and is also identified as increasing one’s risk of overdose, particularly depression [24,25]. Benzodiazepines and a diagnosis of depression have both been found to be associated with receiving high-dosage, compared to low-dosage opioids [26,27,28]. Other conditions identified as increasing overdose risk in opioid analgesic patients includes reduced renal/hepatic function, chronic obstructive pulmonary disorder, sleep-breathing disorders, and substance use [3]. Finally, individuals 65 or older have been identified as a population at risk due to the high incidence of opioid-related adverse events, often attributed to the greater potential for experiencing comorbid conditions or receiving multiple medications that may exacerbate one’s risk of overdose [29,30,31].

Finally, the CDC Guidelines emphasizes the need for clinicians to discuss with patients the risks associated with their opioid therapy, both prior to opioid initiation and periodically throughout their treatment. However, there is little data on the extent to which clinicians engage in these behaviors, particularly when considering the variety of risk factors a patient may present with, which the prescribing healthcare provider may or may not be aware of. A number of barriers that may prevent adequate provider-patient communication such as time, subjective beliefs on the part of the provider that overdose risk may not be large for their opioid-managed patients, and the risk of offending patients due to stigma that links overdose risk with negative perceptions of prescription opioid misuse or being labeled an ‘addict’. Physician communication and engagement with patients may significantly imipact perceptions of overdose risk among their patients and subsequent engagement in risk-management or prevention behaviors.

While much of the concern surrounding overdose risk has, rightfully, centered on individuals receiving opioid medications with a history of use disorder, or identifying those who are engaging in aberrant behaviors with their prescription medications, the CDC Guidelines emphasizes the risk of overdose among patients who adhere to their opioid regimen, which can be complicated by risk factors or being part of a high-risk population (e.g., elderly). However, little is understood about the risks within this patient pool, which makes us the vast majority of those currently receiving opioid medications for chronic pain. The purpose of this study was: (1) to understand the prevalence of various risk factors highlighted by the CDC Guidelines in compliant opioid-managed patients with chronic pain that have already been identified to be at elevated risk for overdose as a result of their opioid dosage level (greater than or equal to 50 daily MME); (2) determine the prevalence of physician communication about overdose risk within this population; and (3) understand patient perceptions of opioid overdose risk. The aim in collecting these data is to provide a greater understanding of the needs for opioid overdose risk mitigation strategies targeted to chronic pain patients who may be at elevated risk for an overdose event stemming from their prescription opioid analgesic therapy.

## 2. Results

### 2.1. Distribution of Daily MME and Concomitant Benzodiazepine Use

Out of 190 CPOM respondents, 73.2% (n = 139) were taking a daily dosage of 50 MME or greater and 52.6% (n = 100) reported concomitant benzodiazepine use. Further delineated into specific sub-groups for analysis, 47.4% (n = 90) were taking 50 MME or greater daily with no benzodiazepine use, 25.8% (n = 49) reported daily MME of 50 or greater with concomitant benzodiazepine use, and finally 26.8% (n = 51) also had concomitant benzodiazepine use, but with a daily dosage less than 50 MME.

**Figure 1:**
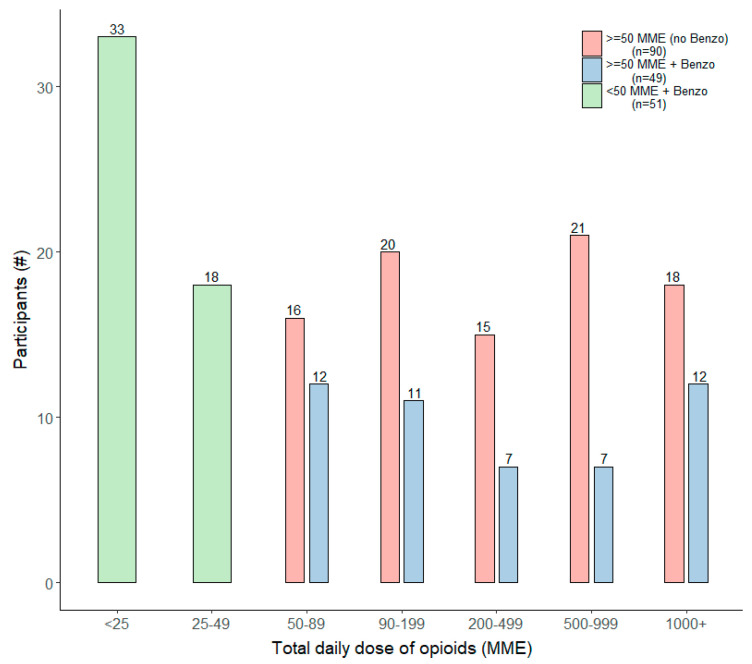
Frequency of daily MME ranges by dosage and concomitant benzodiazepine use groupings.

Figure 1 depicts the frequency distribution of daily MME dose ranges by sub-groups of the CPOM sample. The majority (n = 33) of those with a daily MME of less than 50 and concomitant benzodiazepine use were taking less than 25 MME daily. For those taking 50 MME or greater daily, there was a broad, but fairly even, distribution of dosage levels, with or without benzodiazepine use. Roughly 20% of both groups reported 1000 or more daily MME, while similar proportions reported a daily dosage between 50 and 89 MME. 

### 2.2. Demographics of CPOM Sample

There were several differences identified between sub-groups of the CPOM sample, split out by daily MME ranges and concomitant benzodiazepine use (≥50 MME + No BZD, ≥50 MME + BZD, and <50 MME + BZD). Overall, as shown in Table 1, respondents were primarily white (83.7%–92.2%), female (60.0%–68.6%), and married or living as married (49.0%–63.3%). However, those with ≥50 MME + BZD were significantly younger (mean age 49.9 ± 2.1, *p* < 0.001) than those with ≥50 MME + No BZD (59.6 ± 1.5) or those with <50 MME + BZD (59.1 ± 1.8). Respondents with ≥50 MME and no benzodiazepine use had lower educational attainment, with 32.2% having a high school degree or less, while those with benzodiazepine use attained a college degree or higher (≥50 MME = 53.1%; <50 MME = 43.1%). Employment status also significantly differed, with respondents taking a daily dose of 50 MME or higher being primarily retired if having no benzodiazepine use (52.2%) and primarily employed full-time if there was concomitant benzodiazepine use (44.9%). Those with less than 50 MME per day and benzodiazepine use were split between retirement and full-time employment (39.2 and 29.4%, respectively). Finally, prescription medication was primarily covered by Medicaid Part D in those with <50 MME + BZD (51.0%) and those with ≥50 MME + No BZD (36.7%), while those with ≥50 MME + BZD were primarily covered by private insurance (30.6%) or insurance obtained through work (26.5%).

### 2.3. Prescription Drug Use and Opioid Overdose Risk Factors

As shown in Table 2, CPOM respondents who had a daily MME of 50 or higher had mean daily doses of 596.0 ± 69.5 MME for those without benzodiazepine use and 705.4 ± 129.0 MME for those with benzodiazepine use. The median daily dose for these groups were 325.0 and 301.2 MME, respectively. For those with a daily MME of less than 50 and concomitant benzodiazepine use, the mean daily dose was 20.2 ± 1.7, with a median of 15.0. Hydrocodone was the most frequently prescribed opioid for those with 50 or greater daily MMEs (no benzodiazepine use, 58.9%; benzodiazepine use 65.3%), while tramadol was most frequently reported for those with less than 50 daily MMEs and concomitant benzodiazepine use (41.2%), followed closely by hydrocodone (37.3%). The number of prescription opioids taken daily also significantly differed. While 86.3% of those with <50 MME + BZD took only one prescribed opioid daily, this proportion was significantly smaller in those with 50 or greater daily MME (no benzodiazepine use, 71.1%; benzodiazepine use, 59.2%, *p* < 0.01). There were no significant differences in the benzodiazepines used in the two groups that had concomitant use: alprazolam was the most widely used (39.0%), followed by lorazepam (28.0%) and clonazepam (26.0%).

Disorders identified by the CDC as increasing one’s risk of experiencing an opioid overdose were widely prevalent. Endorsement of at least one risk condition was more widely prevalent in subgroups with concomitant benzodiazepine use, primarily attributable to increased reports of anxiety, depression or other mental health disorder (≥50 MME = 67.3%; <50 MME = 72.5%), compared to those with ≥50 MME + No BZD (33.3%). There were no significant differences in the other risk conditions: 28.9% reported a sleep breathing disorder, 20.0% chronic obstructive pulmonary disorder, and 7.9% kidney or liver disease.

Alcohol use was ubiquitously reported across all groups, with no significant differences,888 and 100% of the total sample endorsing at least one day in the past month where an alcoholic drink was consumed. The majority of the sample (61.8%) reported alcohol use only one day in the past month, with 23.6% having two to four days of alcohol use. Use of alcohol five days or more was reported by 14.6% of the sample.

### 2.4. Perceptions and Provider Communication about Opioid Dosage Level and Risks

Among the entire CPOM sample, less than half (43.2%) reported that their healthcare provider or pharmacist discussed with them the dosage level of their prescribed opioid medication, with no significant differences between sub-groups (Table 3). Respondents with a daily dose of 50 MME or higher primarily perceived their dosage level as “moderate” (no benzodiazepine use, 46.7%; benzodiazepine use, 63.3%), although for those with no benzodiazepine use, 38.9% perceived their dosage level as “low”, compared to 24.5% of those with benzodiazepine use. Those with a daily dose lower than 50 MME and concomitant benzodiazepine use primarily perceived their dosage level as “low” (54.9%), with 29.4% perceiving it as “moderate”.

Communication about risks associated with one’s opioid medication, other than side effects, by one’s provider or pharmacist significantly differed across sub-groups. Those with a daily MME of 50 or greater and concomitant benzodiazepine use had the highest rates of provider communication (73.5%), compared to roughly half by the other sub-groups. There was no significant difference in rates of communication about the risks associated with opioid and benzodiazepine use, which was reported by 64.0% of those taking both opioids and benzodiazepines.

Perceptions of opioid overdose risk did not significantly differ across groups. The majority of individuals in all three groups were aware of the risks of overdose associated with their prescribed opioid medication, with an overall rate of 57.9%. However, 23.2% indicated they did not know whether there was a risk of overdose with their prescribed opioid medication, and 18.9% perceived their medication as having no risk of overdose. Despite awareness of the general risk of overdose associated with their opioid medication, respondents generally did not perceive their individual selves as being at risk of overdose. Nearly two-thirds of the CPOM sample (62.1%) perceived themselves as having no risk whatsoever of experiencing an opioid overdose, with no significant difference across sub-groups. Just 1.6% perceived themselves as high risk of an opioid overdose, with 13.2% perceiving moderate risk, and 23.2% perceiving slight risk. When asked to identify behaviors that increase one’s risk of opioid overdose, less than half (46.8%) identified taking benzodiazepines at the same time as opioids as increasing one’s risk of overdose. Half of the sample identified doubling a dosage after forgetting a previous one as increasing one’s risk (53.2%). Two-thirds of the sample identified the following behaviors as increasing one’s risk of overdose; use of alcohol at the same time as a prescribed opioid (65.8%), using prescribed opioids more often or in larger amounts than directed (68.4%), and taking more than prescribed because of tolerance build-up (68.4%). There were no meaningful differences across sub-groups.

### 2.5. Median Daily MME and Self-Perceptions

**Figure 2:**
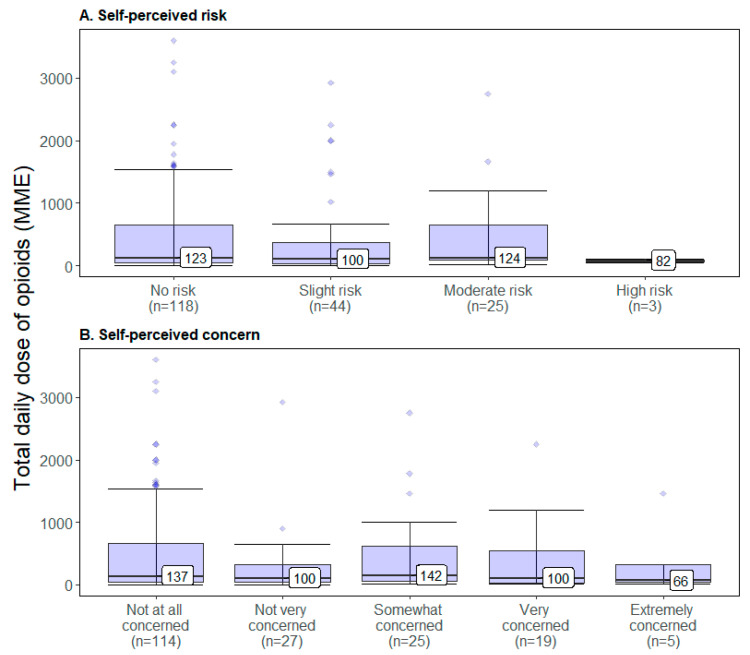
Median daily MME by self-perceived risk (**A**) and concern (**B**) of opioid overdose.

Figure 2 depicts the median daily MMEs by perceived risk and perceived concern of experiencing an opioid overdose across the entire CPOM sample. There was no significant difference in the median daily MMEs across levels of self-perceived risk of experiencing an opioid overdose (*p* = 0.412): those reporting no self-perceived risk had a median of 123 daily MMEs, followed by 100 MMEs for those perceiving slight risk, 124 MMEs for moderate risk and 82 MMEs for high risk. Similarly, there was no significant difference across median daily MMEs across levels of concern that one may experience an opioid overdose (*p* = 0.720): those reporting no concern at all had a median of 137 daily MMEs, followed by 100 MMEs for those who were not very concerned, 142 MMEs for those somewhat concerned, 100 MMEs for very concerned, and 66 MMEs for those extremely concerned.

## 3. Discussion

These data suggest that individuals with chronic pain, prescribed a daily opioid regimen above the CDC Guidelines risk cut-off of 50 MME have a multitude of other risk factors that may additively increase one’s risk of an overdose event. In addition, physician communication was relatively low among this ‘high-risk’ sample, resulting in perceptions among patients that one’s risk of overdose was low or non-existent.

Despite the CDC Guidelines, published in 2016, advocating for a daily opioid dosage of 50 MME or less, 73.2% of the CPOM sample not only exceeded this daily level, but did so to a substantial extent. Roughly 80% of those with ≥50 MME reported daily MME levels above 90, with 20% reporting over 1000 daily MME. However, very few (<10%) respondents perceived their daily dosage as “high”. This discrepancy may reflect the fact that less than half of the sample had a provider or pharmacist discuss their dosage level with them. While for some patients, opioid dosage level was likely to not have been discussed at all by a provider, the discrepancy between perceived dosage, daily reported MME and prevalence of provider discussions suggests that there may be a lack of consistent perception of dosage level at the provider level. Following publication of the CDC Guidelines, there was controversy over the 50/90 MME cut-off points, with a significant amount of disagreement over what constitutes a “high” dosage opioid [32,33]. There is a need for continuing efforts to further determine opioid dosage guidelines that balance the evidence-base, which suggests a dose-response relationship to opioid overdose risk, and individualized, patient-level factors such as tolerance, hyperalgesia, the presence of specific co-morbid conditions, and benzodiazepine utilization. In addition, these topics should be included in educational programming for current and future clinicians that focuses on pain management. Prior research has shown that opioid prescribing behaviors are significantly associated with broader education on pain management, which can be achieved through licensing boards, professional organizations, medical school curricula or continuing medication education (CME) opportunities [34].

While the CDC Guidelines recommend avoiding any concurrent benzodiazepine use alongside prescribed opioids of any dosage, over half of the sample simultaneously used benzodiazepines and their prescription opioid medication. A quarter of the sample appeared to be the most at risk of experiencing an overdose event, using benzodiazepines alongside opioid dosages that had a median of over 300 MMEs daily. Despite reductions in both opioid and concurrent opioid-benzodiazepine prescribing following publication of the CDC Guidelines, prescriptions for both remain high in therapeutic settings within the United States, which is supported by these data from 2020. The risks posed by such high dose opioid prescriptions and concurrent benzodiazepine use is further reinforced by the continuing presence of benzodiazepines in opioid overdose fatalities [18,22]. Despite the high prevalence of benzodiazepine use and associated risk in this sample, over a third of those with concurrent benzodiazepine and opioid use (36%) did not receive any communication from their provider or pharmacist about the potential risks posed by such use, and less than half (46.8%) had knowledge that taking prescribed opioids with benzodiazepines increased one’s risk of overdose. Further research is needed to understand the direct relationship between provider communication and future experiences of opioid overdose events among those receiving both medications. Given the extensive evidence of increased risk, there is a need for interventions (e.g., technological, organizational or systems-based) that not only mandate communication of potential risks when benzodiazepine and opioids are prescribed concurrently, but also provide an opportunity for the provider to ensure that this is the best therapeutic decision for the patient. For instance, a provider who prescribes benzodiazepine to an opioid patient may get an electronic alert through the patient’s electronic health record that provides standardized information to communicate to the patient about the risks associated with the use of benzodiazepines and opioids. In addition, it is possible that these medications are not being provided by the same source. Utilization of prescription drug monitoring programs may provide the opportunity for a clinician to prevent overlapping prescriptions of benzodiazepines and opioids from multiple sources. This may also prevent multiple opioid prescriptions being prescribed to the same patient, which may also increase the risk of overdose. In these data, 30% of the sample reported more than one prescription opioid being taken on a daily basis, reinforcing the need for prescription drug monitoring programs.

The high rate of benzodiazepine use may reflect the prevalence of sleep-related issues (e.g., insomnia) in chronic pain patients, but it is also likely associated with yet another risk factor for opioid overdose, the presence of a mental health disorder, reported here by over half of the sample (52.6%). Chronic pain and mental health disorders are often overlapping conditions, with biological relationships now beginning to be untangled [35], and treatment of both can often be complex. For instance, depression has been found to be significantly associated with increases in opioid dosages, as well as opioid-related adverse events [24,25,26,27,28,36]. Prior mental health issues are also a risk factor for the development of opioid use disorder, as opioids are often perceived by individuals as beneficial in managing mental health issues such as depression, anxiety or experiences of prior trauma. There has been a recent focus on prioritizing mental health in individuals with opioid use disorder, but equally important is the management of mental health issues among those with chronic pain. Most notably, recent evidence has led to warnings about the abrupt/rapid discontinuation or tapering of opioid therapy in chronic pain patients, particularly among those with mental health issues, for which poorer outcomes, including suicidal ideation, have been observed [37]. While further research is needed to understand outcomes associated with tapering and discontinuation, currently there should be an emphasis on caution and individualized care during these processes. Given the intertwined nature of chronic pain and mental health, healthcare settings need to develop interdisciplinary systems of care that can provide opportunities for primary care or pain management physicians to readily and easily screen and refer patients to mental health providers. Use, and mental health are often noted in discussions of opioid risk, we identified the prevalence of other comorbidities which have been shown to increase one’s risk of opioid overdose. While presence of liver or kidney disease was fairly low (7.9%), 20% reported a diagnosis of chronic obstructive pulmonary disorder, and nearly 30% reported a sleep-breathing disorder, which can increase the risk of opioid-related respiratory depression; everyone in this sample reported consuming alcohol on at least one day in the past month. There may be differential effects of alcohol based on the frequency of use, which was not assessed in this study, but given the high prevalence of concomitant benzodiazepine and opioid use, alcohol use should be screened prior to and during a prescribed opioid regimen. In general, there is little information on how often these other, less prominent, risk factors are identified or discussed among clinicians prescribing opioids at high levels or with benzodiazepines; however, given their prevalence in this sample, opioid overdose risk mitigation strategies and prescribing behaviors should take into account as many potential risk factors as possible. Most opioid screeners developed for clinical practice focus on aberrant behaviors or risk of opioid use disorder, but screening for overdose risk factors in medication-compliant patients is an important goal, whether through validated instruments or through algorithms or notifications developed through electronic medical record utilization.

Finally, and importantly, the CDC Guidelines specifically emphasize the need for provider communication focusing on the risks associated with a patient’s opioid therapy, both at the onset of an opioid prescription, and periodically during care. Over 40% of this CPOM sample, at elevated risk for overdose, received no communication from their provider about the risks associated with their opioid medication despite not only high daily dosages of opioids, but a variety of other risk factors present throughout the sample. This likely resulted in the perception among most respondents of this sample that there was absolutely no risk at all of experiencing an opioid overdose, and that these respondents were not at all concerned about such an event occurring. This is a troubling finding given the multitude of risk factors identified, specifically the high prevalence of co-occurring benzodiazepine and opioid use, as well as the fact that this lack of concern or self-perceived risk did not differ by opioid dosage levels. Even the individuals with higher dosages had no corresponding increase in perceived risk or concern. Greater effort is needed to ensure not only that communication about opioid overdose risks is provided by clinicians, but that such discussions result in patient comprehension of these risks. One intervention that may serve to underscore these risks is the co-prescribing of naloxone, a short-acting opioid antagonist used to reverse opioid overdoses. Naloxone co-prescribing has become a primary prevention strategy advocated by numerous governmental, professional and health system organizations, with several states legislating co-prescribing mandates [38,39]. Such provision may not only provide a patient with a potentially life-saving tool, but a secondary effect may be that being co-prescribed naloxone results in greater perception and understanding of one’s risk of opioid overdose. Further research should seek to understand the association between naloxone co-prescribing and patient perceptions of opioid overdose risk.

These data may be limited in their generalization to the larger population of chronic pain, opioid-managed individuals by several factors. For one, our sample was relatively small, sourced from a market research firm, which may not be fully generalizable as respondents had to have access to the internet and the motivation to enroll with the survey platforms. Second, these data are self-reported and not validated by medical records, which may lead to reporting bias in certain areas such as opioid dosage levels or provider communication. Third, the relatively high mean age of the sample (57 years) may also skew population-level inferences; however, given the elevated risk among older populations, this further underscores the risks associated with opioid analgesics. Finally, it was not determined how long these respondents had been undergoing opioid therapy for their chronic pain. It is possible that a certain proportion of these individuals initiated opioid therapy prior to publication of the CDC Guidelines.

## 4. Materials and Methods

### 4.1. Sample Development

A market research firm utilizing two national survey panel companies emailed invitations to the survey to individuals registered with each panel company. The criteria used to define and develop a chronic pain, opioid managed (CPOM) sample identified as being at elevated risk for opioid overdose were based on the Centers for Disease Control and Prevention (CDC) Opioid Prescribing Guidelines, theoretical background, and prior research. Inclusion criteria consisted of the following: (1) 18 years or older; (2) lifetime history of diagnosis or treatment of chronic pain (i.e., pain lasting longer than three months); and, (3) current use of at least one prescription opioid medication to treat chronic pain, continuously, for a period of three months or longer. In this latter instance, “use” was defined as either: a high-dose opioid (i.e., daily dose greater than or equal to 50 morphine milligram equivalents [MMEs], which was the sum of all MMEs, as indicated by respondent identification of current opioid drug(s), dosage, and dosage schedule); or, an opioid at any dose with concomitant use of a benzodiazepine.

Exclusion criteria to minimize bias and the effects of confounding factors included: (1) current or prior employment of a respondent or someone in respondent’s household in the healthcare field; (2) prior history of diagnosis or treatment of alcohol use disorder, opioid use disorder, or other substance use disorder; (3) use in the past three months of illicit drugs (e.g., cocaine, non-prescription amphetamines, barbiturates, non-prescription methamphetamine, heroin, or non-prescription fentanyl); (4) engagement with problematic opioid behaviors (e.g., taking prescription medication more frequently or in higher doses to alter mood or get high; purposeful use of prescription medications with other substances with a goal of altering mood or getting high; taking medication through means other than directed such as chewing, crushing, or parenterally); and, (5) personal experiences with an opioid overdose (e.g., prior opioid overdose; reviving someone who had overdosed on an opioid; knowledge of someone close to the respondent who has overdosed on an opioid).

A total of 7047 CPOM individuals were screened for eligibility, with a total of 190 respondents meeting all inclusion/exclusion criteria and completing a survey from 2 June 2020 to 25 June 2020. Respondents were awarded points to be used through a reward system provided by the survey panel company. All protocols were reviewed and approved by the Washington University in St. Louis Institutional Review Board.

### 4.2. Data Collection and Analysis

With regard to the third inclusion criterion (above), respondents were distinguished by their level of opioid usage—high or low—and the concomitant use of benzodiazepines. To the latter point, any use of benzodiazepines while simultaneously taking one’s opioid medication was considered concomitant use, regardless if it was prescription or non-prescription based, as any use increases one’s risk of opioid overdose. At the time of this survey, 139 respondents (73.2% of the total sample) were receiving a daily dose of opioids ≥50 MMEs, and 100 respondents (62.6% of the total sample) were co-using benzodiazepines at the same time as their opioids. These respondents were further stratified into three mutually exclusive groups to facilitate group comparisons: ≥50 MMEs with no benzodiazepine use (n = 90); ≥50 MMEs, with benzodiazepine use (n = 49); and, <50 MMEs with benzodiazepine use (n = 51).

Descriptive statistics and group-wise comparisons were made across four broad schemas, namely: (1) demographics (i.e., gender; age; race/ethnicity; educational attainment; marital status; employment status; and type of healthcare coverage); (2) the presence and extent of engaging in factors associated with an increased risk of an opioid overdose (i.e., drug dosage; dosage schedule; lifetime comorbid diagnoses/treatment; and number of days in the past month in which alcohol was consumed); (3) perceptions, understanding and knowledge surrounding opioid overdoses and associated risks; and (4) physician/provider communication with respondents about the risks associated with their medications.

Categorical factors were compared using the chi-square test of independence; in some cases, where samples sizes were small (i.e., one cell contained five or less respondents), Fisher’s exact test was utilized instead. All quantitative variables were analyzed and compared according to group variance (i.e., ANOVA). Notably, the aggregate variable, drug dosage, whose mean was often considerably larger than the median—owing to the broad range in respondents’ daily opioid dosage levels—homoscedasticity was estimated using Levene’s (mean-focused) and the Brown–Forsythe (median-focused) test prior to proceeding with the one-way ANOVA; though neither test produced evidence suggesting heteroscedasticity (i.e., unequal variances), the median was reported instead of the mean (see Figure 2A,B).

## 5. Conclusions

In conclusion, these data suggest that opioid overdose risk profiles of chronic pain, opioid-managed individuals are complex and layered with a number of co-occurring risk factors and conditions. However, there were gaps in clinician communication of risks with patients, which may be associated with patient perceptions of having little no to risk of experiencing an opioid overdose. Interventions are needed to identify patients with potential risk factors (e.g., prescription drug monitoring programs, screening tools), implement interventions to improve physician communication (e.g., organizational mandates, educational opportunities) and ensure patient comprehension of these risks, while simultaneously mitigating the reality of such risk (e.g., naloxone co-prescribing).

## Figures and Tables

**Table 1:** Demographics of CPOM sample.

	TOTAL	≥50 MME		BZD	
	(N = 190)	(n = 139, 73.2%)		(n = 100, 52.6%)	
		≥50 MME + No BZD (n = 90, 47.4%)	≥50 MME + BZD (n = 49, 25.8%)	<50 MME + BZD (n = 51, 26.8%)	sig. (χ^2^/F)
Gender					0.735
Female	120 (63.2%)	54 (60%)	31 (63.3%)	35 (68.6%)	
Male	69 (36.3%)	35 (38.9%)	18 (36.7%)	16 (31.4%)	
Non-binary/third gender	1 (0.5%)	1 (1.1%)	0 (0%)	0 (0%)	
Age [Mean (±SE)]	57 (±1.0)	59.6 (±1.5)	49.9 (±2.1)	59.1 (±1.8)	<0.001 *
Race/Ethnicity					0.402
Caucasian or white	165 (86.8%)	77 (85.6%)	41 (83.7%)	47 (92.2%)	
Non-white	25 (13.2%)	13 (14.4%)	8 (16.3%)	4 (7.8%)	
Educational Attainment					
High school graduate or less	46 (24.2%)	29 (32.2%)	7 (14.3%)	10 (19.6%)	0.041 *
Vocational/trade school	13 (6.8%)	4 (4.4%)	5 (10.2%)	4 (7.8%)	0.415
Some college	56 (29.5%)	30 (33.3%)	11 (22.4%)	15 (29.4%)	0.405
College graduate or more	75 (39.5%)	27 (30%)	26 (53.1%)	22 (43.1%)	0.024 *
Marital Status					
Married or living as married	112 (58.9%)	57 (63.3%)	30 (61.2%)	25 (49%)	0.235
Separated or divorced	33 (17.4%)	14 (15.6%)	5 (10.2%)	14 (27.5%)	0.062
Single, never been married	27 (14.2%)	10 (11.1%)	10 (20.4%)	7 (13.7%)	0.323
Widowed	18 (9.5%)	9 (10%)	4 (8.2%)	5 (9.8%)	0.935
Employment Status					
Retired	82 (43.2%)	47 (52.2%)	15 (30.6%)	20 (39.2%)	0.039 *
Full-time employment	58 (30.5%)	21 (23.3%)	22 (44.9%)	15 (29.4%)	0.030 *
Not employed	22 (11.6%)	11 (12.2%)	5 (10.2%)	6 (11.8%)	0.938
Homemaker	17 (8.9%)	6 (6.7%)	6 (12.2%)	5 (9.8%)	0.529
Part-time employment	6 (3.2%)	3 (3.3%)	0 (0%)	3 (5.9%)	0.241
Employment changed recently due to COVID-19	5 (2.6%)	2 (2.2%)	1 (2%)	2 (3.9%)	0.796
Coverage for Prescription Medication					
Medicare Part D	70 (36.8%)	33 (36.7%)	11 (22.4%)	26 (51.0%)	0.013 *
Insurance w/co-pay obtained through work or another organization	44 (23.2%)	21 (23.3%)	13 (26.5%)	10 (19.6%)	0.713
Private insurance-purchased directly from the insurance company	33 (17.4%)	12 (13.3%)	15 (30.6%)	6 (11.8%)	0.017 *
Medicaid	27 (14.2%)	12 (13.3%)	8 (16.3%)	7 (13.7%)	0.884
Purchased all out of pocket/cash	17 (8.9%)	7 (7.8%)	5 (10.2%)	5 (9.8%)	0.864
Prescription discount card	12 (6.3%)	5 (5.6%)	4 (8.2%)	3 (5.9%)	0.824
VA or Military Insurance/Tricare	8 (4.2%)	5 (5.6%)	3 (6.1%)	0 (0%)	0.213

* *p* < 0.05.

**Table 2:** Prescription drug use and opioid overdose risk factors.

	TOTAL	≥50 MME		BZD	
	(N = 190)	(n = 139, 73.2%)		(n = 100, 52.6%)	
		≥50 MME + No BZD (n = 90, 47.4%)	≥50 MME + BZD (n = 49, 25.8%)	<50 MME + BZD (n = 51, 26.8%)	sig. (χ^2^/F)
Mean/Median daily dose of opioids (MME) [Mean (±SE)/Median]	469.7 (±50.7)/120	596 (±69.5)/325	705.4 (±129)/301.2	20.2 (±1.7)/15	<0.001 *
Prescription opioids taken daily for chronic pain					
Hydrocodone	104 (54.7%)	53 (58.9%)	32 (65.3%)	19 (37.3%)	0.010 *
Oxycodone	57 (30%)	32 (35.6%)	15 (30.6%)	10 (19.6%)	0.138
Tramadol	39 (20.5%)	9 (10%)	9 (18.4%)	21 (41.2%)	<0.001 *
Morphine	11 (5.8%)	8 (8.9%)	3 (6.1%)	0 (0%)	0.094
Buprenorphine	9 (4.7%)	4 (4.4%)	4 (8.2%)	1 (2%)	0.339
Hydromorphone	6 (3.2%)	4 (4.4%)	2 (4.1%)	0 (0%)	0.319
Fentanyl	5 (2.6%)	1 (1.1%)	3 (6.1%)	1 (2%)	0.199
Oxymorphone	1 (0.5%)	1 (1.1%)	0 (0%)	0 (0%)	0.572
Other(s)	14 (7.4%)	4 (4.4%)	4 (8.2%)	6 (11.8%)	0.270
Number of prescription opioids taken daily for chronic pain					
One	137 (72.1%)	64 (71.1%)	29 (59.2%)	44 (86.3%)	0.010 *
Two	43 (22.6%)	22 (24.4%)	15 (30.6%)	6 (11.8%)	0.068
Three	6 (3.2%)	1 (1.1%)	4 (8.2%)	1 (2%)	0.064
Four or more	4 (2.1%)	3 (3.3%)	1 (2%)	0 (0%)	0.416
Concomitant benzodiazepine use					
Alprazolam	39 (39.0%)	-	17 (34.7%)	22 (43.1%)	0.387
Lorazepam	28 (28.0%)	-	13 (26.5%)	15 (29.4%)	0.748
Clonazepam	26 (26.0%)	-	15 (30.6%)	11 (21.6%)	0.303
Diazepam)	22 (22.0%)	-	13 (26.5%)	9 (17.6%)	0.284
Clorazepate	3 (3.0%)	-	2 (4.1%)	1 (2%)	0.614
Clobazam	1 (1.0%)	-	1 (2%)	0 (0%)	0.305
Chlordiazepoxide	1 (1.0%)	-	1 (2%)	0 (0%)	0.490
Lifetime history of diagnosis/treatment					
Anxiety, depression, or other mental health disorder	100 (52.6%)	30 (33.3%)	33 (67.3%)	37 (72.5%)	<0.001 *
Sleep breathing disorder (e.g., sleep apnea)	55 (28.9%)	24 (26.7%)	18 (36.7%)	13 (25.5%)	0.374
COPD	38 (20.0%)	16 (17.8%)	12 (24.5%)	10 (19.6%)	0.638
Kidney/liver disease	15 (7.9%)	8 (8.9%)	4 (8.2%)	3 (5.9%)	0.814
Any	128 (67.4%)	47 (52.2%)	39 (79.6%)	42 (82.4%)	<0.001 *
Number of days (in past month) ≥ 1 alcoholic drink was consumed					
One	115 (61.8%)	58 (65.2%)	28 (57.1%)	29 (60.4%)	0.632
Two	19 (10.2%)	6 (6.7%)	9 (18.4%)	4 (8.3%)	0.086
3–4	25 (13.4%)	10 (11.2%)	6 (12.2%)	9 (18.8%)	0.450
5–9	14 (7.5%)	7 (7.9%)	2 (4.1%)	5 (10.4%)	0.490
10–20	7 (3.8%)	2 (2.2%)	4 (8.2%)	1 (2.1%)	0.169
20+	6 (3.2%)	6 (6.7%)	0 (0%)	0 (0%)	0.034 *

* *p* < 0.05.

**Table 3:** Perceptions and provider communication about opioid dosage level and risks.

	TOTAL	≥50 MME		BZD	
	(N = 190)	(n = 139, 73.2%)		(n = 100, 52.6%)	
Dosage		≥50 MME + No BZD (n = 90, 47.4%)	≥50 MME + BZD (n = 49, 25.8%)	<50 MME + BZD (n = 51, 26.8%)	sig. (χ^2^/F)
Pharmacist/healthcare provider talked with you the dosage level of your prescribed opioid medication	82 (43.2%)	34 (37.8%)	26 (53.1%)	22 (43.1%)	0.221
What dose range do you consider your opioid pain medication to be?					
Low dose	75 (39.5%)	35 (38.9%)	12 (24.5%)	28 (54.9%)	0.086 ^†,^*
Moderate dose	88 (46.3%)	42 (46.7%)	31 (63.3%)	15 (29.4%)	0.061 ^†,^*
High dose	11 (5.8%)	6 (6.7%)	4 (8.2%)	1 (2%)	0.742 ^†^
Don’t know	16 (8.4%)	7 (7.8%)	2 (4.1%)	7 (13.7%)	0.493 ^†^
Overdose risk					
Pharmacist/healthcare provider talked with you about the risks, other than side effects, associated with your prescribed opioid medication	111 (58.4%)	46 (51.1%)	36 (73.5%)	29 (56.9%)	0.037 *
Pharmacist/healthcare provider talked to you about the risks associated with taking an opioid and benzodiazepines at the same time	64 (64.0%)	-	34 (69.4%)	30 (58.8%)	0.271
Is your opioid medication for chronic pain associated with a risk for overdose?					
Yes	110 (57.9%)	56 (62.2%)	30 (61.2%)	24 (47.1%)	0.185
No	36 (18.9%)	13 (14.4%)	11 (22.4%)	12 (23.5%)	0.320
Don’t know	44 (23.2%)	21 (23.3%)	8 (16.3%)	15 (29.4%)	0.300
Perceived risk of overdose when using opioids					
No risk	118 (62.1%)	61 (67.8%)	27 (55.1%)	30 (58.8%)	0.289
Slight risk	44 (23.2%)	15 (16.7%)	14 (28.6%)	15 (29.4%)	0.131
Moderate risk	25 (13.2%)	14 (15.6%)	6 (12.2%)	5 (9.8%)	0.609
High risk	3 (1.6%)	0 (0%)	2 (4.1%)	1 (2%)	0.177
Perceived concern of overdose when using opioids					
Not at all concerned	114 (60.0%)	57 (63.3%)	28 (57.1%)	29 (56.9%)	0.673
Not very concerned	27 (14.2%)	14 (15.6%)	6 (12.2%)	7 (13.7%)	0.861
Somewhat concerned	25 (13.2%)	8 (8.9%)	11 (22.4%)	6 (11.8%)	0.073
Very concerned	19 (10.0%)	8 (8.9%)	4 (8.2%)	7 (13.7%)	0.579
Extremely concerned	5 (2.6%)	3 (3.3%)	0(0%)	2 (3.9%)	0.401
Which of the following increases your risk of overdosing from an opioid?					
Taking more than prescribed at one time because one built up a tolerance and needed more for it to be effective	130 (68.4%)	58 (64.4%)	35 (71.4%)	37 (72.5%)	0.531
Using prescribed opioids more often or in larger amounts than directed or prescribed by a healthcare provider	130 (68.4%)	61 (67.8%)	35 (71.4%)	34 (66.7%)	0.863
Use of alcohol at the same time as a prescribed opioid	125 (65.8%)	57 (63.3%)	33 (67.3%)	35 (68.6%)	0.788
Taking someone else’s prescribed opioid	106 (55.8%)	53 (58.9%)	25 (51%)	28 (54.9%)	0.664
Forgetting to take a prescribed dose and then doubling up the next time	101 (53.2%)	50 (55.6%)	26 (53.1%)	25 (49%)	0.756
Taking prescribed opioids in combination with benzodiazepines	89 (46.8%)	40 (44.4%)	26 (53.1%)	23 (45.1%)	0.597

* *p* < 0.05; ^†^ statistics restricted to ≥50 MME individuals only (n = 139).

## Data Availability

Data is contained within the article.
